# The surgical management of obesity with emphasis on the role of post operative imaging

**DOI:** 10.2349/biij.7.1.e8

**Published:** 2011-01-01

**Authors:** S Smith, F Hampson, M Sinclair

**Affiliations:** 1 Department of Radiology, Addenbrooke’s Hospital, Cambridge, United Kingdom; 2 Department of Hepatobiliary and Upper GI Surgery, Ipswich Hospital, Suffolk, United Kingdom; 3 Department of Radiology, Ipswich Hospital, Heath Road, Suffolk, United Kingdom

**Keywords:** Bariatric surgery

## Abstract

The role of surgery in the morbidly obese is becoming more prominent. There are a variety of surgical approaches which can be used and radiology plays a crucial role in post operative follow up, particularly in the management of complications. Many general radiologists remain unfamiliar with both the normal and abnormal appearances after bariatric surgery and this pictorial review aims to bridge this gap.

## INTRODUCTION

In recent years, we have witnessed an obesity epidemic with a high prevalence in many countries [1–4]. In association with this explosion, there has been a significant increase in the number of patients seeking a surgical solution to their problems. Patients are termed obese when their body mass index (BMI) ie, weight (kilograms)/ height (metres)^2^, exceeds 30. Adults whose BMI exceeds 40 are potential candidates for surgery. Motivated patients with acceptable operative risks who are considered to have a low probability of success with non surgical measures, as demonstrated for example by failing dietary or exercise regimes, may be considered for surgery. In certain cases, less severely obese patients (with BMI between 35 and 40) may also be considered for surgery. Included in this category are patients with high-risk co-morbidities such as severe diabetes mellitus, sleep apnoea and obesity related cardiomyopathy [5].

The aim of bariatric surgery is to reduce caloric intake by either restricting the amount of calories an individual can take in or reducing the amount of calories absorbed from the GI tract. The most common types of bariatric surgery are Roux-en-Y gastric bypass (RYGBP) [6] (restrictive and malabsorptive), Gastric banding (restrictive) [7] and biliopancreatic diversion (BD) with duodenal switch (malabsorptive and some restrictive) [8]. During the course of this review, we will focus on these techniques. Other procedures include Vertical Banded Gastroplasty and Gastric pacing [9]. Vertical Banded Gastroplasty is now only rarely performed because of the inferior weight loss compared with RYGBP [10] and the high rate of late complications [11].

The aforementioned procedures can all be associated with a number of complications which can be subtle and difficult to diagnose at an early stage. Given the limited physiologic reserve of the patients involved, it is essential that complications are identified early and managed appropriately. It is, therefore, important that the radiologist has an understanding of the anatomical and functional changes that result from these procedures and the potential complications unique to each. Close interaction with the surgical team is also essential.

## ROUX-EN-Y GASTRIC BYPASS

The concept of gastric bypass to aid weight loss was introduced by Mason and Ito in 1967 [12]. The operation they described has evolved into the Roux-en-Y gastric bypass. This is now the most frequently performed type of bariatric surgery in North America. It may be performed using an open or a laparoscopic approach. This procedure has a number of advantages. For instance, the majority of type 2 diabetics become euglycaemic. This is thought to relate to the exclusion of the passage of nutrients through the duodenum [13, 14]. In addition, early delivery of nutrients to the hindgut aids weight loss by causing malabsorption. The combined restrictive and malabsorptive effects result in increased weight loss and a greater success rate compared with gastric banding, despite the increased operation time and complication rates [15].

## PROCEDURE

**Figure 1:**
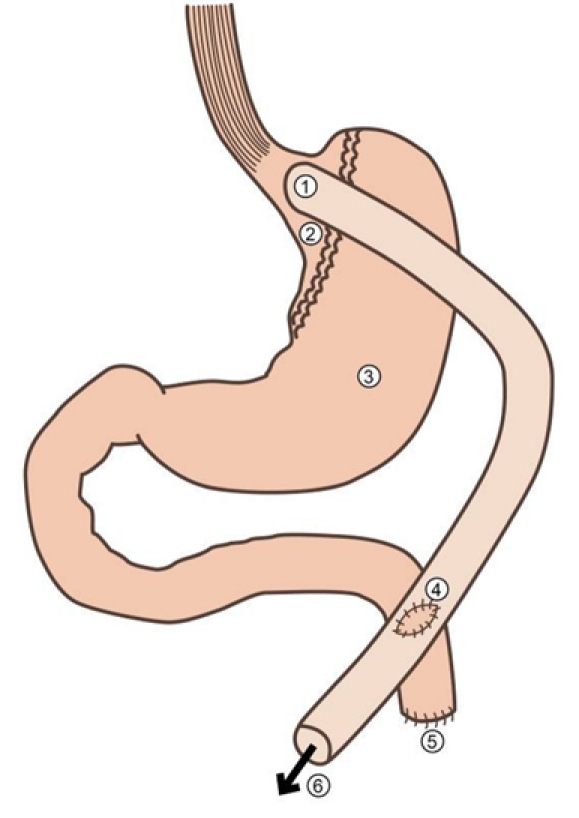
Schematic representation of bariatric gastric bypass procedure (1: gastro-jejunostomy, 2: remnant gastric pouch proximal to staple line, 3:defunctioned distal stomach, 4: jejunojejunostomy, 5:closed end of defunctioned jejunum, 6: To distal jejunum).

In general, the proximal stomach is partitioned to create a gastric pouch of 30 ml or less in volume [16]. The pouch is constructed from the gastric cardia with the exclusion of the acid-producing fundus. Having been excluded from the enteral flow, the gastric remnant is left *in situ*. The proximal jejunum is then used to create a Roux limb. A gastrojejunostomy is then formed via a side-to-side anastomosis between the gastric pouch and the distal limb of jejunum. Bowel continuity is finally restored by the formation of a jejunojejunostomy between the Roux loop and the biliopancreatic limb (Figure 1). Distension of the small gastric pouch results in early satiety, and exclusion of the duodenum and proximal jejunum results in malabsorption.

## NORMAL IMAGING FINDINGS POST ROUX-EN-Y GASTRIC BYPASS SURGERY

Both upper gastrointestinal (UGI) contrast studies and computed tomography (CT) are useful in displaying the normal postoperative anatomy and aid the detection of complications. An UGI contrast series is usually performed routinely within 48 hours after surgery [17, 18]. Narrowing of the gastrojejunostomy with delayed pouch emptying in the early postoperative period may be misinterpreted as anastomotic stenosis, but in the majority of cases it is simply a sign of normal post operative oedema [18]. Contrast medium in the jejunal limb should demonstrate normal mucosal folds and motility [19].

**Figure 2:**
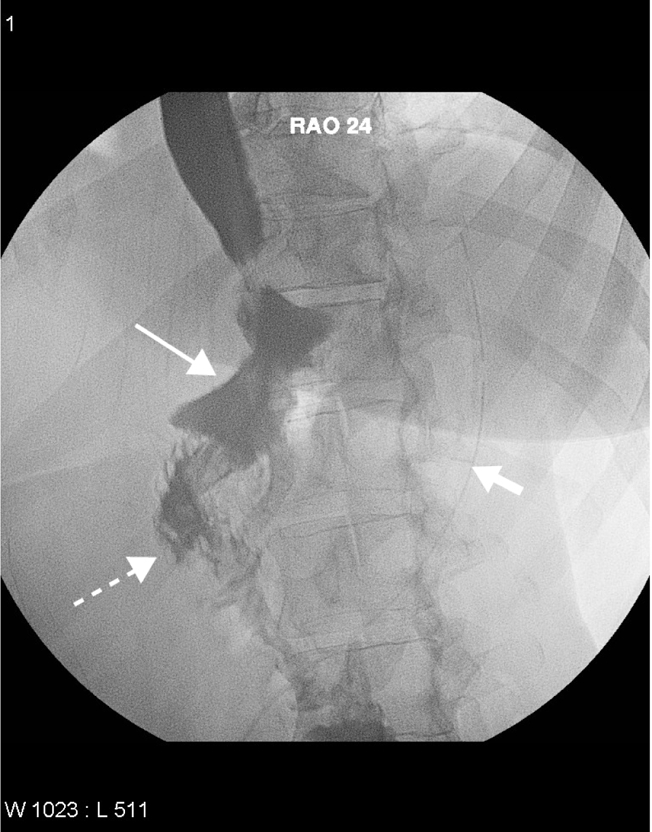
Normal upper gastrointestinal barium study post bariatric gastric bypass. Showing surgical drain (short arrow), gastric pouch (long arrow), jejunum distal to gastro-jejunostomy (hashed arrow). Note the normal jejunal fold pattern.

A gastric pouch volume of approximately 30 ml and a 12 mm diameter outlet stoma are thought to yield the best results [18]. While it is not possible to quantify the volume of the gastric pouch on conventional radiographs, it should be a size similar to that of a lower thoracic or lumbar vertebral body [18] (Figure 2). The distal side-to-side anastomosis is difficult to visualise because contrast material rarely refluxes from the jejunum into the pancreaticobiliary limb [20]. Similarly, the duodenum and the excluded stomach are difficult to visualise.

**Figure 3:**
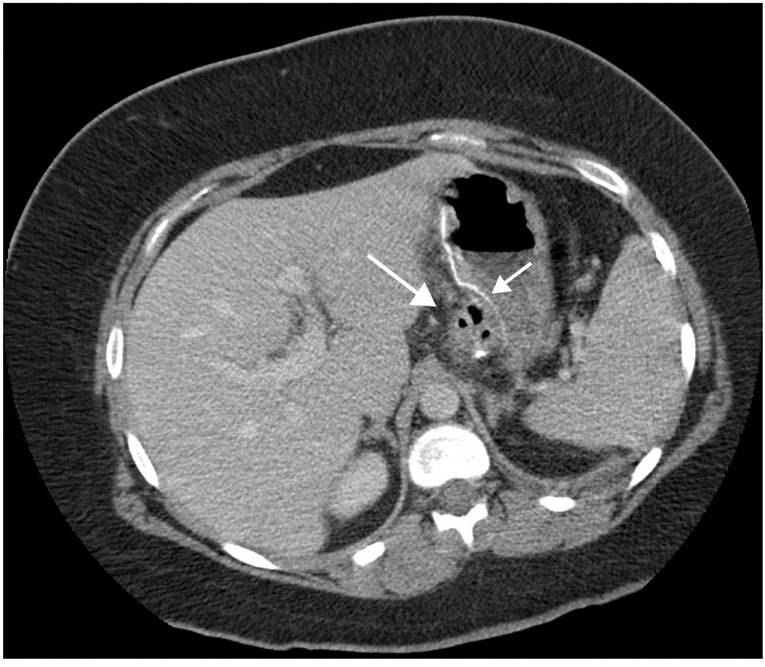
Normal upper abdominal CT scan with intravenous contrast medium. Gastric pouch (long arrow), staple line crossing the gastric body and defunctioning the distal stomach (short arrow). Note the undistended gastric remnant and normal calibre small bowel.

CT offers a detailed view of the anatomy, including all important structures that are not clearly demonstrated on the UGI contrast series. A normal gastric pouch is usually collapsed at the time of CT. The blind loop and the Roux limb should not be larger than 2.5 cm in diameter [18]. The roux limb can usually be followed either retrocolically or antecolically to the distal anastomosis with the pancreaticobiliary limb [18]. The excluded stomach may contain a small amount of air and fluid, but it should never be distended. The native duodenum and the proximal jejunum (part of the pancreaticobiliary limb) can also be followed to the lower anastomosis and should not be greater than 2.5 cm in diameter [18] (Figure 3(a) and (b)).

Despite its advantages over the UGI contrast series, the cost and additional radiation burden of CT limit its routine application in the immediate postoperative period. It is “the gold standard” when complications are suspected, or have been demonstrated on the initial imaging.

## IMAGING THE COMPLICATIONS OF ROUX-EN-Y GASTRIC BYPASS SURGERY

The complications of RYGBP can be divided into those which occur early (within one month of surgery), and those which occur late (more than one month after surgery) in the postoperative period. Early complications include anastomotic leaks and abscess formation, dilated excluded stomach and ileus [21]. Late complications include stomal stenosis, marginal ulcer formation, and incisional, non incisional ventral and internal hernia formation [21]. Staple line dehiscence and bowel obstruction may present during either period [21].

## ANASTOMOTIC LEAK

Extra luminal anastomotic leakage is the most feared early complication following RYGBP. It has an incidence of 1% - 6% and is usually diagnosed within ten days following surgery [20, 22–28]. It occurs most frequently at the gastrojejunal anastomosis [20, 29]. Leakage occurs less commonly from the distal oesophagus, gastric pouch, blind-ending jejunal limb, or, rarely from the jejuno-jejunal anastomosis. If not recognized early and treated promptly, it is a potentially lethal complication [17, 27, 30, 31]. Repeat surgery is required in up to 80% of cases [31]. Up to 75% of post operative leaks result in left upper quadrant fluid collections [31]. These are most frequently perisplenic and may evolve into abscesses [20]. Extra luminal leaks may also cause peritonitis and chronic fistula formation [17, 27, 31, 32].

**Figure 4:**
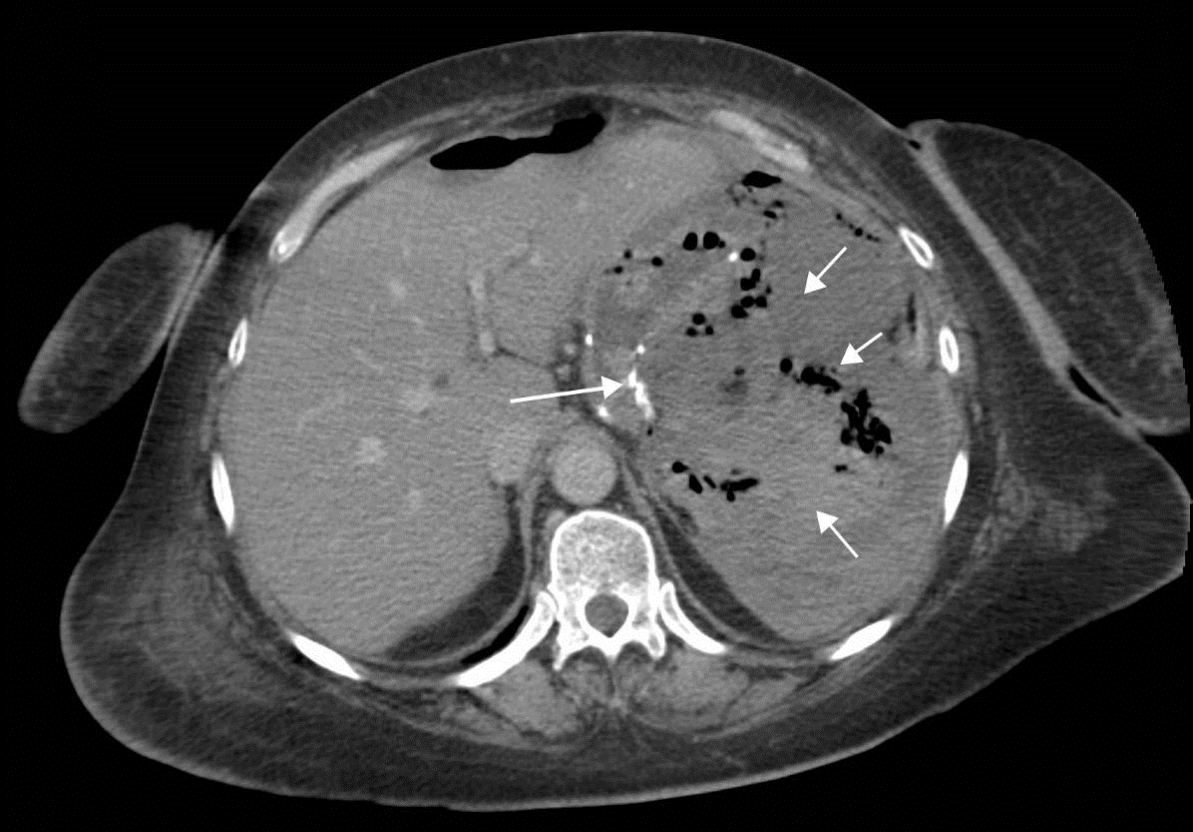
Upper abdominal CT scan with IV contrast enhancement 10 days post bypass procedure, showing a large irregular abscess containing gas and semi-solid material in the upper abdomen (short arrows). Note the upper margin of the gastric staple line (long arrow). The collection was drained percutaneously but a further laparotomy was required to repair a leak at the gastro-jejunostomy site.

UGI contrast studies are useful in the assessment of extra luminal leaks. Contrast medium may be seen to flow into the peritoneal cavity. It is important to note that a leak may seal off after a fluid collection has formed and may, therefore, elude detection. Patient body habitus may also compromise image quality and, hence, limit the radiologist’s ability to detect the specific site of leakage. CT, therefore, plays an important role in the detection of both leaks and fluid collections and can also be used to guide drainage and potentially obviate the need for surgery [29] (Figure 4).

In order to aid accurate interpretation of these studies, it is important to be aware of the circumstances that may mimic a free leak. These include the presence of plication defects which are focal outpouchings and irregularities along the suture lines. There are several factors which help to prevent the misinterpretation of plication defects as free leaks. Firstly, plication defects readily fill and empty with contrast medium and secondly, they have well-defined margins and often contain gastric rugal folds.

Contrast may also leak across the gastric staple line into the excluded stomach. This form of leakage is not associated with the increased morbidity and mortality of a free leak. An UGI contrast study may demonstrate a small collection of contrast material extending to the left of the gastrojejunal anastomosis. Importantly, normal gastric rugal folds can be identified within such a collection. Contrast medium may also enter the gastric remnant via retrograde flow most often in the presence of an ileus or a distal obstruction. Under these circumstances, contrast will only be seen in the left upper quadrant in the region of the gastrojejunal anastomosis on delayed imaging allowing differentiation from a free leak. Differentiation may also be assisted by the presence of contrast medium within the duodenum and the excluded limb [17].

## GASTRIC STAPLE LINE DEHISCENCE

Gastric staple line dehiscence may occur in the early or late postoperative period. It permits communication between the small gastric pouch and the excluded stomach. It may, therefore, result in inadequate weight loss and ultimately failed RYGBP surgery [33]. It has been reported to occur in up to 3% of patients [26, 34]. Early dehiscence may be the result of inadequate division of the gastric pouch at surgery. On the other hand, it may be the result of a free leak [17]. In the late postoperative period, it is thought to be due to over distension of the gastric pouch with food [17, 26, 32].

**Figure 5:**
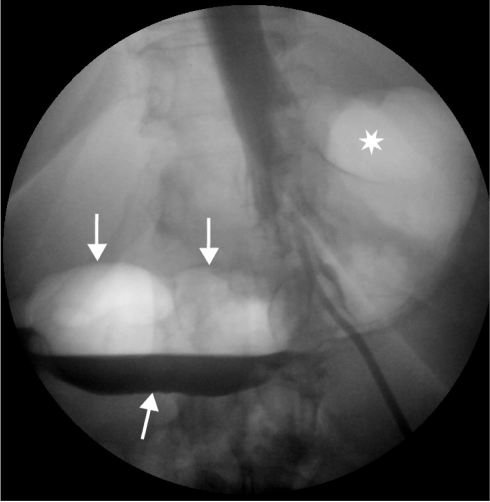
Upper gastrointestinal contrast study performed several weeks after bypass surgery. The gastric staple line has broken down and contrast enters the defunctioned stomach (arrows). Note the gas filled fundus/gastric pouch (asterix).

Appearances on an UGI series, the imaging modality of choice, depend upon the degree of dehiscence. Contrast medium may preferentially enter the excluded stomach with little or no opacification of the jejunal limbs (Figure 5). Alternatively, there may be preferential flow through the gastrojejunal anastomosis with very little contrast medium entering the excluded stomach. It is important to note that contrast medium may enter the excluded stomach via retrograde flow and, hence, diagnosis should be made on initial examination rather on delayed views. This makes the diagnosis of gastric staple line dehiscence difficult on CT examination. The presence of contrast medium within the fundus of the excluded stomach, in the absence of opacification of the more distal excluded stomach and duodenum is suggestive of dehiscence [21].

## ANASTOMOTIC STENOSIS

Anastomotic stenosis is most likely to occur at the gastrojejunal anastomosis where it is reported to have an incidence of up to 10% [20, 22, 25, 29, 34]. It is the result of fibrosis which is thought to be secondary to ischaemic change and usually occurs more than one month following surgery with a mean of 49 days [17, 20, 22, 25, 29, 34, 35]. Patients typically present with postprandial epigastric pain, vomiting and excessive weight loss. UGI examination demonstrates a dilated oesophagus and gastric pouch with delayed emptying. There may be associated oesophageal dysmotility. The pouch is commonly spherical, contains an air-fluid-contrast level and may contain a large amount of debris [32]. Endoscopic dilatation is the treatment of choice with a success rate of up to 95% [35]. Stenosis at the jejuno-jejunal anastomosis is much less common with a reported incidence of 0.9% [17, 20]. Stenosis at this level frequently requires surgical revision, particularly if the anastomosis cannot be visualised endoscopically [32].

## SMALL BOWEL OBSTRUCTION

Small bowel obstruction occurs in up to 5% of cases [24, 29, 30, 34, 36–41]. Early postoperative obstruction may be caused by oedema and/or haematoma, and usually resolves spontaneously [26, 32, 33]. This most commonly occurs at the proximal or distal anastomosis but in the presence of a retrocolic gastrojejunal anastomosis there is often oedema and/or haematoma at the site where the Roux jejunal limb crosses the transverse mesocolon [21].

Adhesions and internal hernia formation are other major causes of small bowel obstruction. The prevalence of adhesion formation has been reduced by using a laparoscopic approach [25, 34] although this has led to an increase in the prevalence of internal hernia formation [34, 42]. Adhesions are more common during the first month following surgery, whereas internal hernia formation usually occurs later [34]. Bezoar formation in the gastric pouch and intussusception have also been reported [29, 30, 43, 44]. Intussusception most commonly occurs at the jejuno-jejunal anastomosis with the anastomotic suture line acting as a lead point [21, 43].

Internal hernia formation following RYGBP surgery has been reported in up to 3% of cases [25, 29, 34, 42]. If a retrocolic path is chosen for the Roux limb, two potential internal hernia spaces are created. The first (and most common) is at the transverse mesocolon where the Roux limb passes through the mesenteric window. The second is the Petersen’s defect that arises between the mesentery of the Roux limb and the base of the mesentery of the transverse colon. Another potential space occurs between the mesenteries of the Roux and biliopancreatic limbs at the jejunojenunostomy. This defect is present regardless of the path taken by the Roux limb. Care is taken to close these defects [16]. The herniated bowel is usually the Roux limb itself with a varying amount of additional small-bowel loops [29]. The associated symptoms may be intermittent and non-specific [42]. Volvulus, infarction and perforation may result, especially if diagnosis and treatment are delayed. Awareness of this potentially devastating complication is, therefore, essential.

It is difficult to differentiate between small-bowel obstruction caused by adhesions and that caused by an internal hernia on UGI studies and CT. Both can result in a fixed appearance of the small bowel. However, adhesions are usually associated with an angulated, tethered appearance rather than a clustered appearance. An abnormal cluster of dilated small bowel loops in the left upper or mid abdomen is highly suggestive of an internal hernia [45–47]. The clusters are relatively fixed, remaining high even in the upright position. There is usually associated stasis within the clustered small bowel loops. In addition, there may be a visible loop of bowel entering and exiting the clustered segment [21]. Crowding and congestion of the mesenteric vessels are also common features [29].

The clustered loops of bowel often displace other parts of the bowel. When herniation occurs through the transverse mesocolon, the cluster of dilated bowel loops is located posterior to the stomach upon which it may exert mass effect. When it occurs through the small bowel mesentery, the cluster is pressed against the abdominal wall with no overlying omental fat, causing central displacement of the colon [29]. Herniation through the Peterson’s defect is more difficult to identify given that it has neither a confirming sac nor a characteristic location [20]. It is important to remember that urgent surgical intervention is mandatory regardless of the underlying cause of small bowel obstruction.

An important consequence of distal postoperative obstruction is acute distension of the excluded stomach. Natural decompression is not possible and gastric perforation or leakage from the gastrojejunal anastomosis may result in the absence of prompt treatment which may involve percutaneous needle decompression or gastrostomy catheter insertion [21].

## MARGINAL ULCERS

Marginal ulcers have a reported incidence of up to 3% following RYGBP. They occur around the gastrojejunal anastomosis, most frequently in the jejunum adjacent to the anastomosis [30, 34]. Their exact aetiology is uncertain. The most widely supported theory is that they are the consequence of exposure of the jejunal mucosa to gastric secretions. There is indeed a decrease in incidence with decreased gastric pouch size and, hence, decreased gastrin and acid production [32]. They also respond well to medical treatment with proton pump inhibitors and antibiotics if the patient is *Helicobacter pylori* positive. Another theory is that the ulceration is the result of ischaemia, although with a peak incidence in the second postoperative year this seems unlikely. Finally, it is possible they may be caused by reflux of bile. This should not, however, be a common occurrence if the Roux limb is of adequate length [16].

The consequences range from mild epigastric pain and chronic anaemia to frank haemorrhage requiring urgent intervention. Chronic perianastomotic ulceration may also cause stricture formation necessitating endoscopic dilatation or surgical revision. Fistula formation between the pouch and the excluded stomach is another reported complication [16].

Diagnosis is usually made via endoscopy. Detection may be difficult on UGI studies and nearly impossible on CT [48]. On UGI examination, marginal ulcers appear as small focal out-pouchings of contrast medium at or adjacent to the gastrojejunal anastomosis. There is stasis within the ulcer crater and frequently adjacent mucosal fold thickening and oedema [21].

## ACUTE DILATATION OF THE GASTRIC REMNANT

**Figure 6:**
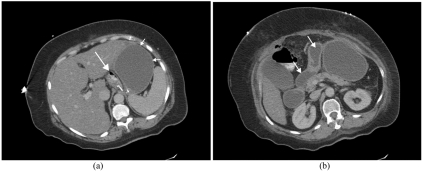
CT examination of the upper abdomen with IV and oral contrast. Acute dilatation of the distal gastric remnant in a patient presenting with severe upper abdominal pain and electrolyte imbalance two weeks after bypass surgery. (a) Dilated distal defunctioned stomach (short arrows), collapsed gastric pouch (long arrow) which contains some oral contrast. (b) Image inferior to (a), dilated duodenum (white arrow) and proximal jejunum (white arrow). This settled with conservative management.

This usually occurs in the immediate post surgical period and presents with upper abdominal pain and fullness. If severe, it can lead to significant constitutional upset but it usually settles with conservative management. The diagnosis is made easily on CT examination (Figure 6).

## LIVER METABOLIC CHANGES

**Figure 7:**
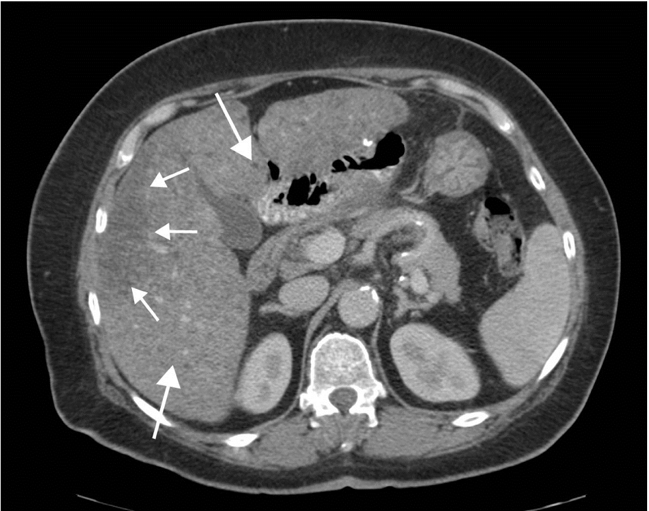
Upper abdominal CT examination in a patient approximately three months after bariatric gastric bypass, performed for upper abdominal pain. There are abnormal liver appearances with multiple small well defined areas of low attenuation (long arrows) together with a larger more confluent area with a typical ‘geographical’ appearance peripherally in the right lobe (short arrows). These changes were due to patchy fatty infiltration.

Fatty change is commonly seen in the liver on follow-up imaging. This presumably reflects the abrupt change in nutrient absorption, which occurs in the postoperative period. Local experience is of occasional gross liver change, which can mimic other disease processes. There is often a rise in the liver function tests, which tend to normalise without specific intervention (Figure 7).

## LAPAROSCOPIC ADJUSTABLE GASTRIC BANDING

A restrictive gastric banding procedure was first introduced in 1983. By 1986, the bands were made adjustable [49] and a laparoscopic approach was made available in the early 1990s [50, 51]. Laparoscopic adjustable gastric banding (LAGB) is easier to perform and has lower complication rates than RYGBP and BD [52–54]. However, it may not be as successful in the long term, especially in those whose BMI exceeds 50 [16, 53, 55–58].

## PROCEDURE AND BAND ADJUSTMENT

Prior to LAGB, an UGI study should be performed in order to evaluate the anatomy, assess oesophageal motility and determine if there is a hiatus hernia [59–61]. Oesophageal motility disorders and fixed hiatus hernias may be associated with increased postoperative complications such as band slippage and dysphagia [60, 61].

LAGB involves placing a silicone band around the upper stomach to create a small gastric pouch (approximately 15 ml in volume) [62] and a narrow stoma (approximately 12 mm in diameter) [59, 63] which communicates with the rest of the stomach. The serosa proximal and distal to the band is sutured in order to cover the anterior portion of the band and prevent slippage [60, 63, 64]. Before the bands were made adjustable, poor weight loss was seen in those whose stoma was too large and dysphagia and/or obstruction in those whose stoma was too small. These problems were negated by the introduction of the adjustable gastric band, which allows percutaneous adjustment of the banding device without the need for further surgery.

The silicone band has an adjustable inner balloon cuff that is connected by tubing to a subcutaneous injection reservoir that is usually sutured to the anterior rectus sheath. The diameter of the band may, therefore, be increased and the stoma narrowed by injecting the port with saline or water-soluble contrast medium. Aspiration deflates the cuff and widens the stoma. The band system is left empty after surgery [59, 60, 65]. Adjustments are performed around six weeks postoperatively, once oedema has resolved [48, 49, 55]. UGI examination is performed before and after the adjustments in order to ensure adequate stoma size and the absence of obstruction [56].

Adjustments are usually performed under fluoroscopic guidance by the radiologist following consultation with the surgeon [59–61, 65, 66, 68]. Having located the centre of the subcutaneous port, the radiologist places a radiopaque marker on the skin. Following skin preparation and infiltration of local anaesthetic, a 20- to 22-gauge, non coring, deflected tip needle is used to access the port [60, 65]. Use of a non-coring needle helps to prevent damage to the port and leakage from the system. Puncture of the tubing rather than the port may also cause leakage and device failure [60]. Adjustments may also be made under ultrasound guidance. It is important to remember that an UGI examination is still required following ultrasound-guided band adjustment [68].

## IMAGING THE COMPLICATIONS OF LAGB

LAGB is generally considered to be a safe procedure [67]. Some degree of morbidity may occur in up to 35% [69]. Additional surgery may be necessary in 11% [51, 56–58, 63, 69–72]. There is minimal perioperative mortality [56, 60, 63, 66]. Regurgitation and gastroesophageal reflux are common until dietary habits are adjusted [65]. Early complications are otherwise rare. These include gastroesophageal perforation [56, 57, 63, 65, 70], inappropriate band placement [65], early band slippage [53, 56, 63, 70] and acute stomal stenosis [72].

Late complications are much more common, particularly pouch dilatation and band slippage [65, 66, 69, 70, 73]. Other potential late complications include intragastric band migration or erosion and device-related complications resulting from leakage or infection [56, 57, 63–66, 70, 72–76].

UGI contrast studies and CT are the primary imaging techniques used in postoperative evaluation. Use of multiplanar reconstruction with multi detector CT enables delineation of the gastric band and any slippage or migration [62].

## ACUTE GASTRIC PERFORATION

Gastric perforation occurs in 0.1–0.8% of cases [53, 57, 59, 77, 78]. The clinical presentation is varied. While patients usually present with abdominal pain and pyrexia, less obvious signs of sepsis such as tachycardia and anxiety may be the only manifestations. CT is the investigation of choice. Evidence of perforation and abscess formation may be detected [62].

## STOMAL STENOSIS AND POUCH DILATATION

Acute stomal stenosis may result from blockage of the stoma by food or postoperative oedema. Patients present with nausea, vomiting, and upper abdominal pain. UGI studies demonstrate slow passage of contrast medium and narrowing of the stoma. Assuming the gastric band is appropriately sited and deflated, the management is conservative [62].

Failure to comply with dietary advice may lead to chronic concentric pouch dilatation with a normal or widened stoma [65, 66]. Weight loss may cease and it may necessitate removal of the gastric band. While chronic pouch dilatation occurs in up to 25% of cases [76], the incidence usually ranges from 3% to 8% [59, 79].

Concentric pouch dilatation may also occur if the stoma is too narrow. This is more likely to occur acutely as a result of over inflation of the gastric band. Patients present with symptoms of obstruction. Acute pouch dilatation may also be due to a focal weakness within the band with eccentric herniation and associated eccentric stomal narrowing. This can be readily identified by filling the band with contrast medium at fluoroscopy [67]. Different projections may be required to appreciate it during an UGI study [65].

When pouch dilatation is the result of a narrow stoma, the band should be deflated immediately [59]. If deflation is delayed, pouch dilatation will recur despite deflation in up to 50% of patients and may be irreversible [59]. This is the result of a tissue reaction to the silicone band which causes perigastric fibrosis [62].

## GASTRIC BAND SLIPPAGE

Gastric band slippage occurs in up to 24% of patients [53, 54, 61, 63, 72, 80–82]. It may be caused by overeating and overfilling of the gastric pouch, over inflation of the gastric band, recurrent vomiting or faulty surgical technique [74, 83, 84]. The incidence has decreased considerably with surgical modifications [52, 63, 65, 66, 68, 74, 76, 82–86]. The adoption of appropriate dietary habits is also important [76].

**Figure 8:**
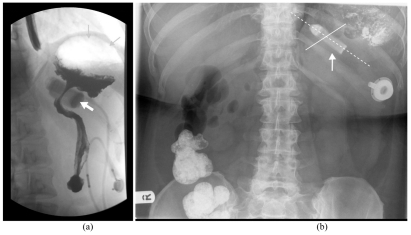
Examples of distal band movement. (a)Upper GI contrast study after gastric banding. The band is readily identified (short arrow) and has slipped inferiorly around the body of the stomach. The proximal stomach is too capacious (long arrows) (b) Plain abdominal film sometime after a contrast study shows the band (arrow) is in an abnormal location. The axis of the band (as indicated by a hashed lie) has rotated and lies in a ‘twenty past ten’ position rather than the more normal ‘ten past eight’ (solid line).

**Figure 9:**
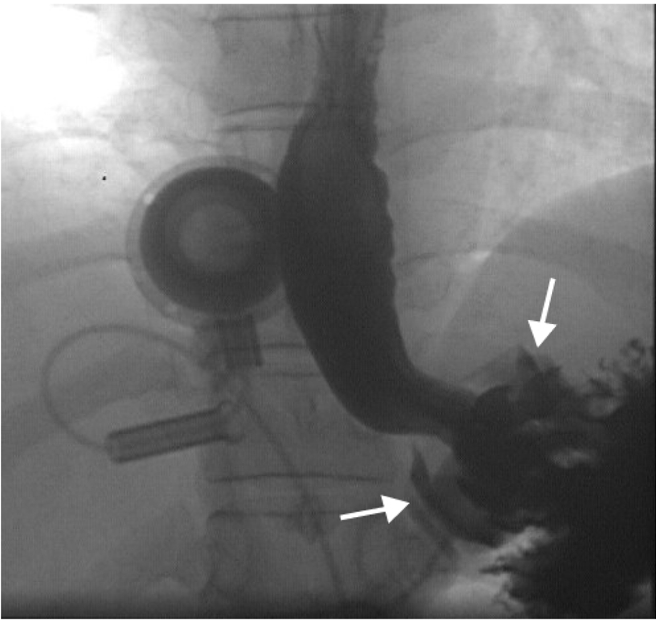
Upper GI contrast study after gastric banding showing rapid transit past the band (arrows) indicating that it is too loose.

**Figure 10:**
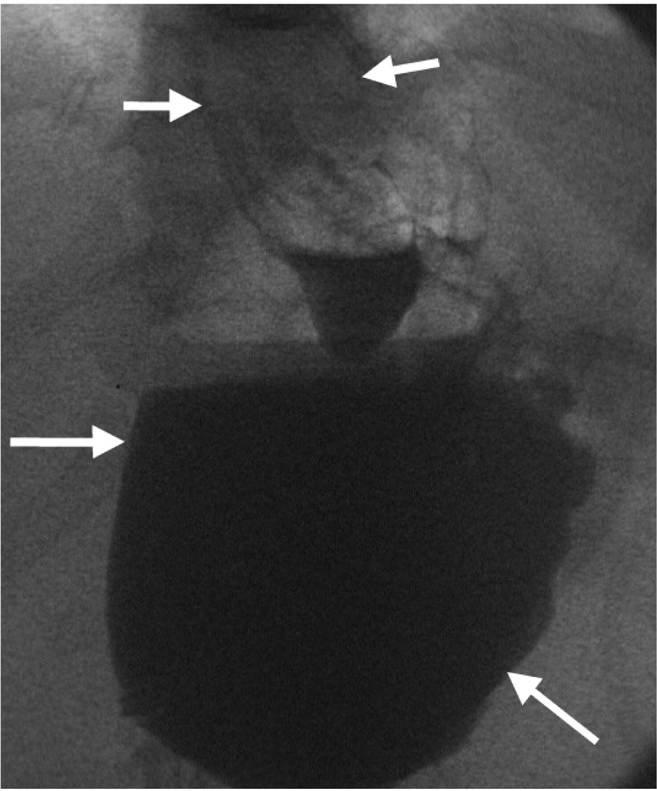
Upper GI contrast study showing slight oesophageal dilatation (short arrows) and filling of a very large viscus (long arrows). At surgery these appearances were due to band migration and volvulus of the dilated proximal stomach. This was surgically corrected.

The two most common types of slippage are anterior and posterior slippage. Rarely, concentric slippage occurs with complete displacement of the band distally [80]. They all present in a similar fashion but have different radiological findings. Posterior slippage is associated with upward herniation of the posterior stomach wall through the band [62]. Anterior slippage is related to insufficient fixation of the band at surgery [67]. This enables the higher pressure in the upper pouch to push the band downwards over the anterior aspect of the stomach [62]. With anterior or posterior slippage, UGI contrast studies demonstrate the band to be in a more vertical or horizontal position with associated eccentric pouch dilatation [27, 34, 37, 54]. The pouch is usually posterior and inferior in posterior slippage and anterior and superior in anterior slippage [62] (Figures 8, 9, 10).

Band slippage may be asymptomatic. Patients may, however, present with food intolerance, epigastric pain, vomiting, progressive gastroesophageal reflux, oesophageal motility disorders or early satiety [57, 80, 83]. Intermittent slippage may also cause chronic eccentric pouch dilatation and subsequent weight gain [65]. The band may slip back into a normal position following emptying of the gastric pouch or deflation of the band [65]. In a minority of cases, band slippage may produce acute gastric obstruction, gastric volvulus, focal gastric ischaemia, gastric infarction, perforation and haemorrhage [65, 84]. Gastric necrosis is a rare, life threatening complication of band slippage that may occur years after band placement [83, 86]. This complication often necessitates total gastrectomy [86]. Early detection of band slippage is, therefore, essential if the associated complications are to be avoided. The band should be completely deflated when slippage has been diagnosed [60, 83]. Repositioning or replacement of the band is then required [67].

## INTRAGASTRIC BAND EROSION OR MIGRATION

The reported prevalence of intragastric band erosion or migration, in which the silicone ring penetrates the gastric wall and in some cases the lumen of the stomach, varies from 0–11% [64, 87]. The prevalence appears to increase over time [67]. It may be related to intra operative damage to the outer gastric wall, the use of non-steroidal anti-inflammatory drugs, excessive vomiting, over inflation of the band or band infection [63, 65].

Clinical manifestations may include non-specific abdominal pain, cessation of weight loss, abdominal and/or port site abscess, perforation and peritonitis [64, 65, 87–89]. Fatal gastrointestinal haemorrhage may also occur. In order to avoid such complications, urgent surgical removal of the band and repair of the stomach is required [64, 65, 87–89].

UGI studies cannot detect band erosion in its early stages [90]. The findings are, however, pathognomonic later on when contrast medium is seen to surround the part of the band that has migrated into the gastric lumen. The band, therefore, appears as an intraluminal filling defect [64, 65]. It is important to note that there may be no other sign of perforation. CT is advised if symptoms suggest intra abdominal sepsis or open perforation [64].

## DEVICE-RELATED COMPLICATIONS

Device-related complications have been reported in 1.4–26% of patients [55, 69, 70, 91, 92]. This variation is in part due to the length of follow up. Potential complications include infection, system leakage resulting in band deflation, and migration or inversion of the reservoir preventing band adjustments [67]. Rotation or inversion of the reservoir is more common following major weight loss [63].

The subcutaneous reservoir can become infected and an abscess may form [62]. Diagnosis may be hampered by the patients’ body habitus. CT or ultrasound examination may, therefore, be required in order to make the diagnosis. The use of fluoroscopy and the proper technique to perform adjustments reduces the number of failed punctures and, hence, decreases the risks of damage and infection [92].

Leakage of saline from the system with associated band deflation may occur in up to 5% of patients and usually requires surgical repair [59, 63, 65, 74, 92, 93]. In the majority of cases, leakage occurs from the reservoir [67]. It may also occur from the connecting tubing as a result of accidental puncture during adjustment [59, 63]. Alternatively, the tubing may become disconnected [67].

If leakage is suspected, plain radiography may demonstrate acute angulation or disconnection of the connecting tube [59, 74]. Leakage from the system may be further assessed by injecting a selected volume of saline into the reservoir and measuring the return volume on deflation. A reduction in volume implies a leak [92]. It is important to distinguish leakage from the reservoir from leakage from the band or tube, because leakage from the reservoir may be corrected by a simple surgical procedure under local anaesthetic, whereas a leaking band or disconnected port must be removed and replaced [62, 93]. Injection of water-soluble contrast medium into the reservoir under fluoroscopy helps to determine the exact site of leakage [93].

There are a number of other reported complications associated with the connecting tubing. These include intra-abdominal sepsis and abscess formation, wound infection with enterocutaneous fistula formation and subsequent erosion of the tubing into the bowel, disconnection of the tubing with migration into the small bowel, and intracolonic penetration by the tubing [73, 94, 95].

## BILIOPANCREATIC DIVERSION

The increasing number of patients presenting for surgery in the superobese category has focused attention on more extreme malabsorptive procedures for weight loss [16]. Currently, there are three techniques that permit weight loss beyond that routinely achievable by the standard “short limb” RYGBP. They are Biliopancreatic diversion (BPD), BPD with duodenal switch, and the very long limb RYGBP [16].

## PROCEDURE

BPD is a malabsorptive procedure. It involves an improvised distal horizontal gastrectomy (residual stomach with a capacity varying between 200 and 400 ml) with a 200–300 cm long alimentary/ Roux limb which facilitates fat malabsorption [16, 96, 97]. The gastric pouch is, therefore, larger than that created in the RYGBP procedure. It must be larger in order to accommodate the patient’s need for more protein and calorie supplementation in order to prevent malabsorptive-induced malnutrition.

A modification of BPD that has gained some popularity in North America is the duodenal switch procedure [98–100]. The BPD is modified by first replacing the distal gastrectomy with a sleeve resection of the stomach. This results in the creation of a lesser curve gastric tube. The proximal duodenum is divided just distal to the pylorus (preserving the pylorus and a few centimetres of duodenum helps to prevent dumping and marginal ulceration). The small intestine is then manipulated creating a short length alimentary limb that will connect as a Roux limb just distal to the pylorus. The majority of the remaining small bowel remains connected to the defunctioned biliary limb which is anastomosed to the roux limb 50–100 cm proximal to the ileocaecal valve. This creates a common channel where food may mix with digestive juices [101].

## COMPLICATIONS OF BPD

Symptoms of malabsorption, such as diarrhoea, bowel frequency, dumping, and flatulence, are more prominent following BPD than after standard RYGBP. Other potential complications include protein calorie malnutrition, bone demineralisation and infection [16]. Surgical complications include incisional hernia formation, anastomotic leak, fistula formation, intestinal obstruction and marginal ulceration.

## INTESTINAL OBSTRUCTION

The incidence of intestinal obstruction following BPD is 1–5% [102–104]. Presenting symptoms depend on the site of obstruction. The typical symptoms and radiological signs of bowel obstruction will be present if the alimentary limb is obstructed but not when the biliopancreatic limb is obstructed. Diagnosis of biliopancreatic limb obstruction is, therefore, more difficult. Patients present with vague abdominal pain and nausea. Prompt diagnosis with the aid of CT and surgical intervention is required in order to prevent hepatic and pancreatic damage, and when possible, to preserve the postoperative anatomy [105].

## MARGINAL ULCER FORMATION

Marginal ulcer formation is most common in the first postoperative year following BPD [105]. Prophylactic histamine antagonists or proton pump inhibitors, and abstinence from smoking and alcohol reduce the incidence [102, 104, 106]. Stomal ulcers tend to produce periumbilical pain rather than the epigastric pain typically associated with gastroduodenal ulcers [107]. Complications include anaemia and stomal stenosis. Emergency surgical complications are rare. They include massive haemorrhage and perforation. The duodenal switch procedure helps to prevent marginal ulceration.

## CONCLUSION

With an increasing number of patients undergoing surgery for morbid obesity, it is vital that radiologists have an understanding of the most commonly performed techniques and their unique complications. Early recognition of these complications is essential and close interaction between the radiologist and the surgical team will help to optimize outcomes in this demanding patient group.
